# Substance use and related problems among U.S. women who identify as mostly heterosexual

**DOI:** 10.1186/s12889-015-2143-1

**Published:** 2015-08-20

**Authors:** Tonda L. Hughes, Sharon C. Wilsnack, Arlinda F. Kristjanson

**Affiliations:** Building Interdisciplinary Research Careers in Women’s Health (BIRCWH), University of Illinois at Chicago, College of Nursing (M/C 802), 845 S. Damen Ave Room 1160, IL 60612-7350 Chicago, U.S; Department of Psychiatry and Behavioral Science, University of North Dakota, School of Medicine & Health Sciences, 501 North Columbia Road Stop 9037, 58202-9037 Grand Forks, ND, U.S

## Abstract

**Background:**

We used data from a nationally representative sample to compare substance use outcomes among adult women who identified as mostly heterosexual with those who identified as exclusively (only) heterosexual.

**Method:**

We analyzed data from mostly heterosexual women and only heterosexual women in Wave 5 (2001) of the National Study of Health and Life Experiences of Women (weighted *n* = 1085).

**Results:**

Mostly heterosexual women were significantly more likely than only heterosexual women to report every alcohol-related outcome included in our analyses except lifetime treatment. Odds of lifetime and past-year marijuana and cocaine use showed larger differences, with mostly heterosexual women nearly four times as likely as only heterosexual women to report lifetime cocaine use and five times as likely to report past-year use.

**Conclusions:**

We recommend that researchers use measures of sexual identity that include more nuanced response options, and that health care providers learn about the existence, large numbers, and risk/protective factors associated with substance use patterns of mostly heterosexual women.

## Background

Women who identify as “mostly heterosexual” make up the largest single group of sexual minority women in studies that allow for this response option; in fact, they are generally far greater in number than are lesbian and bisexual respondents combined. For example, in Wave 3 of the National Longitudinal Study of Adolescent Health 10.3 % (*n* = 762) of women aged 18–27 identified as mostly heterosexual compared with 2.5 % (*n* = 187) of women who identified as bisexual and 1.1 % (*n* = 84) who identified as mostly or exclusively lesbian [1]. Similarly, among 25–30-year-old respondents in the nationally representative Australian Longitudinal Study of Women’s Health, 6.4 % identified as mainly heterosexual whereas 1.1 % identified as bisexual and 1.1 % as mainly or exclusively lesbian [2].

Despite mostly heterosexual women’s large numbers, the majority of studies that ask about sexual identity do not provide the intermediate response option of “mostly heterosexual” (or “mostly lesbian/gay”); rather, they typically use questions that ask respondents to identify as heterosexual/straight, bisexual or lesbian/gay. In a methodological experiment conducted with a large random sample of undergraduate college students in a midwestern U.S. university, McCabe and colleagues examined responses and study outcomes based on whether response options were the commonly used 3-category sexual identity questions (heterosexual/straight, bisexual, lesbian/gay), or an expanded 5-category question that also included the intermediate options of mostly heterosexual and mostly lesbian/gay [3]. One-third of students who identified as bisexual in response to the 3-category question chose mostly heterosexual or mostly lesbian/gay in response to the 5-category question. In addition to demonstrating that the 3-category question can result in misclassification of sexual identity for a substantial number of persons, McCabe et al. also found that it resulted in different findings related to study outcomes. Specifically, using exclusively heterosexual as the comparison category, bisexual respondents showed the greatest risk of substance use in analyses using the 3-category measure, whereas mostly heterosexual respondents showed the greatest risk when data were analyzed using the 5-category measure.

Other studies of adolescent or young adult samples that have included the intermediate “mostly” response option have also found that, compared with their exclusively heterosexual counterparts, female and male respondents who identify as mostly heterosexual show elevated risks of a number of negative health outcomes, including risky drinking and use of illicit substances and tobacco use [1, 4–8]. In addition, several of these studies add support to a growing body of literature suggesting that substance use-related health disparities are greater across subgroups of sexual minority *women* than across subgroups of sexual minority men [6, 8, 9].

Although researchers have demonstrated the value of assessing a broader range of sexual identities in studies of youth, very little is known about the health or health risk behaviors of *adult* women who identify as mostly heterosexual [10, 11]. Only a handful of studies of adult women have examined differences between mostly and exclusively heterosexual identity [2, 12–14]. A recent review of the literature on psychological and physical health of male and female mostly heterosexuals identified 60 papers that used data from 22 samples [15]. Of the 22 samples represented, only 5 (one being the NSHLEW used in the present paper) included adult women in all stages of the adult life cycle (i.e., not children, adolescents, college students, or young adults only). Among these studies of adult women, still fewer have assessed differences in substance use outcomes in women who identify as mostly heterosexual.

To address this gap in the literature we used data from a national probability sample of adult women—the National Study of Health and Life Experiences of Women [16]—to compare substance use outcomes among women who identified as mostly heterosexual with those who identified as exclusively heterosexual. Our primary hypothesis was that alcohol and drug use, and their adverse consequences, would be higher among women who identified as mostly heterosexual than among those who identified as exclusively (only) heterosexual.

## Methods

### Sample

For the current analyses we used existing data from the 2001 survey of the National Study of Health and Life Experiences of Women (NSHLEW). The sample is a national probability sample of U.S. women aged 21 and older, followed longitudinally between 1981 and 2001. Women who reported consuming 4 or more standard drinks in a week were oversampled. Respondents were interviewed at 5-year intervals. Completion rates were consistently high (≥80 % in most waves and subgroups). Attrition analyses showed that women lost to follow-up were older and less educated than women who were re-interviewed, but the two groups did not differ in their drinking patterns. In all surveys, statistical weights adjusted for the oversampling of heavier-drinking women and for variations in response rates. The weighted 2001 sample was demographically similar to adult women in the 2000 U.S. Census, with the exceptions that more women in the NSHLEW were classified as non-Hispanic white and fewer women reported less than a high school education.

Of the 1126 women (weighted N) in the 2001 sample, 1044 (92.7 %) reported being only heterosexual; 48 respondents (4.3 %) reported being mostly heterosexual. There were too few women who identified as bisexual (*n* = 10), mostly lesbian (*n* = 3), only lesbian (*n* = 7), or “other” (*n* = 9) to include them in the current analyses. Five women did not respond to the sexual identity question.

### Fieldwork

Fieldwork for the survey was conducted by staff of the National Opinion Research Center (NORC), University of Chicago. Data were collected in the fall to minimize seasonal variations and to avoid the increased drinking typical of the winter holiday season. Face-to-face interviews conducted by female NORC interviewers averaged 75–90 min in length. Interviewers received extensive training in general interviewing techniques and study-specific training focused on potentially sensitive topics, including substance use, sexual experience, and sexual orientation. Interviews were conducted at a location chosen by the respondent – usually her home – with no family members or other persons present. Potentially sensitive topics, such as physical and sexual abuse and sexual identity, were located toward the end of the interview, when rapport was well established.

The interview included questions about drinking behavior, drinking-related problems, and numerous possible antecedents and consequences. Questions and scales originally were selected from well-validated instruments, with some alcohol-related questions modified to increase their sensitivity to characteristics of women’s drinking. Respondents provided informed consent prior to each interview. All survey methods and procedures were reviewed and approved by the Institutional Review Boards of the University of North Dakota and the National Opinion Research Center.

### Measures

The primary question about *sexual identity* asked, “Recognizing that sexuality is only part of your identity, how do you define your sexual identity?” Response categories were: only heterosexual, mostly heterosexual, bisexual, mostly homosexual or lesbian or gay, and only homosexual or lesbian or gay. This and other questions related to sexual orientation were developed in two focus groups with Chicago-area sexual minority women [17] and pilot tested in a small pilot study of 120 lesbian and heterosexual women.

The interview included a number of measures of s*ubstance use and its consequences*. Alcohol-related measures included (1) heavy drinking (consumption of ≥2 standard drinks [≥1 oz. ethanol] per day, based on estimates that combined information about beverage-specific drinking frequency, quantity, typical drink size, and ethanol content for beer, wine, and distilled spirits); occurrence in the past 12 months of (2) any intoxication (“drinking enough to feel drunk—where drinking noticeably affected your thinking, talking and behavior”), (3) any heavy episodic drinking (≥6 drinks in a day), (4) any problem consequence (of 8 consequences, including driving a car while high from alcohol, and drinking-related problems with partner, children, job, or housework), and (5) any symptom of potential alcohol dependence (of 5 symptoms, including inability to stop drinking before becoming intoxicated, and inability to stop or cut down on drinking over time); (6) ever being concerned about having a drinking problem; and (7) ever receiving treatment for a drinking problem. Drug-related measures included (1) ever smoking tobacco; (2) smoking tobacco in past 12 months; (3) any lifetime marijuana use; (4) marijuana use in past 12 months; (5) any lifetime cocaine use; and (6) cocaine use in past 12 months. Additional information about these measures, and about sample design and survey procedures, can be found elsewhere [16, 18].

### Data analysis

Sample weights for the *total* sample (see Methods, paragraph 2) adjusted for the oversampling of heavier drinking women and were further adjusted to reflect the actual number of women in the total sample. Because our analyses included just two sexual identity groups – only heterosexual women and mostly heterosexual women – the weights were again readjusted slightly so that the total weighted subsample n (only heterosexuals plus mostly heterosexuals) equaled the actual number of women in these two groups. We used IBM SPSS Version 21 to construct cross-tabulation tables for demographic characteristics of the subsample. SAS 9.4 was used to conduct logistic regression analyses with Firth’s bias correction, thereby producing penalized maximum likelihood estimates [19, 20].

Separate logistic regression models were tested to examine the relationship of sexual identity to each outcome of interest while controlling for age, race/ethnicity, and education.

## Results

Demographic characteristics of the only heterosexual and mostly heterosexual women are shown in Table 1. Mostly heterosexual women were significantly younger than only heterosexual women, but the distribution of race/ethnicity and education did not differ significantly between the two groups.

**Table 1:** Demographic characteristics of only heterosexual and mostly heterosexual women

	Only Heterosexual	Mostly Heterosexual	TOTAL
N (unweighted/weighted)	1013/1037	72/48	1085/1085^a^
	Weighted Percentage^b^		*p*
Age Group			<.001
21–34	25.5 %	52.1 %	
35–49	31.2 %	29.2 %	
50–64	23.0 %	14.6 %	
65+	20.3 %	4.2 %	
Race			ns
White	72.4 %	85.4 %	
Black, African American	17.0 %	10.4 %	
Hispanic, Latina	8.6 %	4.2 %	
Asian/Pacific Islander	0.8 %	0.0 %	
American Indian	0.6 %	0.0 %	
Other	0.8 %	0.0 %	
Education			ns
College degree or higher	22.4 %	27.1 %	
Some college or less	77.6 %	72.9 %	

^a^Weight adjusted to reflect total unweighted N

^b^Some percentages total more than 100 % due to rounding

As shown in Table 2, rates for the four illicit drug use variables were higher among mostly heterosexual women than among only heterosexual women. Odds of lifetime marijuana use (OR = 2.75, CI_95%_:1.49, 5.08) and marijuana use in the past 12 months (OR = 3.25, CI_95%_: 1.54, 6.86) were substantially higher among mostly than only heterosexual women, as were odds of lifetime (OR = 3.53, CI_95%_: 1.27, 9.78) and past-12-month (OR = 4.57, CI_95%_: 1.01, 20.66) cocaine use. The two groups did not differ on lifetime or current smoking.

**Table 2:** Substance use behaviors among only heterosexual and mostly heterosexual women

	Only Heterosexual	Mostly Heterosexual	TOTAL
N (unweighted/weighted)	1013/1037	72/48	1085/1085^a^
	Weighted Percentages		OR^b^ (CI._95_)
Smoking, ever	49.2 %	64.6 %	1.61 ns (0.86, 3.01)
Smoking, currently	21.3 %	27.1 %	0.94 ns (0.47, 1.87)
Marijuana use, ever	17.4 %	43.8 %	2.75** (1.49, 5.08)
Marijuana use, past 12 months	6.1 %	25.0 %	3.25** (1.54, 6.86)
Cocaine use, ever	2.5 %	10.4 %	3.53* (1.27, 9.78)
Cocaine use, past 12 months	0.8 %	6.3 %	4.57* (1.01, 20.66)
Heavy drinking, past 12 months	3.8 %	14.6 %	4.28** (1.76, 10.44)
Intoxication, past 12 months	26.2 %	54.2 %	1.92* (1.01, 3.65)
Heavy episodic drinking, past 12 months	12.7 %	31.3 %	2.02* (1.03, 3.94)
Drinking-related problem consequences, past 12 months	12.3 %	31.3 %	2.22* (1.13, 4.33)
Symptoms of dependence, past 12 months	9.1 %	31.3 %	3.08** (1.55, 6.10)
Ever concerned about having a drinking problem?	17.9 %	39.6 %	2.55** (1.38, 4.70)
Since last interview, ever concerned about having a drinking problem?	6.5 %	20.8 %	2.78** (1.30, 5.97)
Ever had treatment for a drinking problem?	3.8 %	10.4 %	2.54 ns (0.95, 6.83)

^a^Weight adjusted to reflect total unweighted N

^b^Odds Ratio controlling for age, race/ethnicity (other vs. white), and education (some college or less vs. college degree or higher)

*p < .05; **p < .01; ***p < .001

We found statistically significant sexual identity differences on every alcohol-related outcome variable, with the exception of seeking alcohol treatment. Mostly heterosexual women were more likely than only heterosexual women to report 12-month heavy drinking (OR = 4.28, CI_95%_: 1.76, 10.44), drinking to intoxication (OR = 1.92, CI_95%_: 1.01, 3.65), and heavy episodic drinking (OR = 2.02, CI_95%_: 1.03, 3.94). They were also significantly more likely to report drinking-related problem consequences (OR = 2.22, CI_95%_: 1.13, 4.33) and symptoms of potential alcohol dependence (OR = 3.08, CI_95%_: 1.55, 6.10). Similarly, mostly heterosexual women were significantly more likely to report that, at some point in their lifetime, they had wondered or worried about whether they were developing a drinking problem (OR = 2.55, CI_95%_: 1.38, 4.70), as well as to report this concern in the previous five years (OR = 2.78, CI_95%_: 1.30, 5.97).

Because same-sex attraction has been shown to be associated with negative substance abuse outcomes among adolescent sexual minority women, and same-sex behavior has been associated with negative substance use outcomes among both adolescent and adult sexual minority women [21–23], we also compared these sexual orientation dimensions in women with mostly heterosexual and only heterosexual sexual identities (Table 3). Whereas 75 % of mostly heterosexual women reported some same-sex attraction (compared with 5 % of only heterosexual women), only 28 % of mostly heterosexual women who were ever sexually active reported any same-sex behavior (compared with 2 % of only heterosexual women).

**Table 3:** Gender of sexual partners and sexual attraction among only heterosexual and mostly heterosexual women

	Only Heterosexual	Mostly Heterosexual	TOTAL
N (unweighted/weighted)	1013/1037	72/48	1085/1085^a^
	Weighted Percentage		p
Gender of Partners			<.0001
Only men	85.4 %	66.0 %	
Mostly men	1.0 %	19.1 %	
Men and women equally	0.0 %	2.1 %	
Mostly women	0.0 %	0.0 %	
Only women	0.5 %	4.3 %	
Not sexually active	13.2 %	8.5 %	
Attraction			<.0001
Only men	94.7 %	24.5 %	
Mostly men	4.4 %	55.1 %	
Men and women equally	0.9 %	18.4 %	
Mostly women	0.0 %	0.0 %	
Only women	0.0 %	2.0 %	
	Only Heterosexual	Mostly Heterosexual	TOTAL
	Weighted Percentages		OR^b^ (CI._95_)
Same-sex partners^c^	3.7 %	29.8 %	10.97 (5.26, 22.91)***
Same-sex attraction	5.3 %	75.0 %	46.17 (22.08, 96.57)***

^a^Weight adjusted to reflect total unweighted N

^b^Odds Ratio controlling for age, race/ethnicity (other vs. white), and education (some college or less vs. college degree or higher)

^c^Analysis excluded women who were never sexually active

***p < .001

## Discussion

Although several studies have compared substance use outcomes among heterosexual women who report same-sex sexual partners with heterosexual women who do not, few have made these comparisons based on sexual identity (thought to be the most important dimension of sexual orientation, predicting many health outcomes)—primarily because only a handful of studies have included the intermediate “mostly” identity option [24–26]. Our findings of higher rates of hazardous alcohol use, and higher rates of lifetime and current marijuana and cocaine use, among mostly heterosexual women are consistent with findings from younger populations (adolescents and college students) and from the small number of studies that have included adult samples [15]. These findings suggest that most studies of adult SMW’s drinking have likely misclassified this substantially large group of women who, when analyzed separately, appear to be at elevated risk of engaging in heavy or problematic alcohol and other drug use.

In an effort to better understand the characteristics of women who identify as mostly heterosexual, and how they may differ from only heterosexual women, we compared the two groups on sexual attraction and sexual behavior. Although mostly heterosexual women in our study were significantly more likely than only heterosexual women to report both same-sex attraction and any same-sex sexual partners, the magnitude of the group difference in same-sex attraction was substantially larger than that for same-sex partners. These findings support the assertion that same-sex behavior is not a critical component of identifying as mostly heterosexual [27]. Indeed, same-sex *attraction* may be a more reliable indicator, given that sexual *behavior* is influenced by many factors (e.g., social approval, religious belief, opportunity) other than sexual orientation.

### Why are mostly heterosexual women at higher risk for substance use/misuse?

Two frequent explanations for health disparities in sexual minority populations are (1) minority stress, and (2) risks of a nonheterosexual lifestyle [15]. The minority stress explanation [28] highlights the excess of stressors (e.g., discrimination, victimization, rejection, internalized biphobia/homophobia) experienced by sexual minorities due to the stigma of non-heterosexuality in heterosexist societies. The combination of sexual-minority-specific stressors with the general stressors that all people face results in excessive and chronic stress, which may lead to health-risk behaviors (such as substance abuse, overeating, smoking) in an attempt to cope. The non-heterosexual lifestyles explanation would focus on high-risk characteristics of sexual minority lifestyles and environments, for example, the traditional importance of the gay bar as a primary venue for social interaction [29]. However, because women who identify as mostly heterosexual tend to view themselves and be viewed by others as heterosexuals rather than sexual minorities rarely affiliate with same-sex communities, and rarely have same-sex partners [10, 27], it is unclear how helpful these explanations of sexual minorities’ health disparities are in understanding mostly heterosexual women’s elevated rates of alcohol and drug misuse.

Like lesbian women or gay men who are not “out”, mostly heterosexual women might be described as having “concealable stigmatized identities” (CSIs), identities that might be socially devalued and stigmatized if known, but in individuals with no obviously stigmatizing attributes [30]. The factors contributing to substance abuse or other health risk behaviors may be quite different in persons with CSIs than in persons with non-stigmatized identities or persons whose stigmatized identities are obvious to others (e.g., racial/ethnic minorities, openly gay or lesbian individuals with same-sex partners, or persons with physical abnormalities) [31]. For example, preoccupation with the CSI, fears of discovery, and beliefs about the negative consequences of discovery can trigger cognitive processes, such as thought suppression, that paradoxically can lead to intrusions and heightened awareness of the suppressed thoughts, resulting in increased psychological distress, depression, and anxiety which the individual attempts to escape via substance use or other health risk behaviors.

Related to the cognitive, affective, and behavioral consequences of CSIs proposed by Talley and Littlefield [31], additional sources of psychological distress for mostly heterosexual women may be feelings of uncertainty or ambiguity about their sexual identity because it deviates from the commonly recognized trichotomy of heterosexual, bisexual, or lesbian/gay, and a sense of sexual identity confusion resulting from the lack of congruence among the major aspects of one’s sexuality (identity, behavior, and attraction) [32].

Meyer [28] describes the importance of both individual and community-level social support mechanisms in coping with minority-based stressors. For mostly heterosexual women attempting to cope with psychological distress resulting from their concealed stigmatized identity, however, such support from others similar to themselves may be difficult to find. Like bisexual identity, mostly heterosexual identity deviates from the norm—both in the larger society and in sexual minority communities. In a recent qualitative study of bisexual women, participants reported social interactions with both heterosexuals and sexual minorities that questioned the reality of their bisexuality, denied or dismissed the legitimacy of their sexual identity, portrayed them as sexually indiscriminate, or blatantly denigrated bisexuality and bisexual persons [33]. Similarly, Bower, Gurevich and Mathieson [34] found that bisexual participants reported being treated as indecisive, in transition, or using their identity as a ploy to retain heterosexual privilege. Bisexual women who look to the lesbian and gay community for support and safe haven are often met with suspicion and even rejection. Mostly heterosexual women, even more than bisexual women, are invisible and lack an identifiable community or support system. Thus, to the extent that mostly heterosexual women experience CSI-related psychological distress, they lack an identifiable support community of persons like themselves that might be able to help buffer and reduce this distress.

### Limitations

Several limitations of this study relate to its sample and to its sexual identity question. First, although researchers, clinicians and people in the general population prefer clear-cut and familiar labels, people do not always fit precisely into the labels they are provided. In addition, terms used to describe sexual identity, like all language, are imperfect and evolving. Providing more nuanced labels such as ‘mostly lesbian’ and ‘mostly heterosexual’ gives women greater flexibility in describing who they are without having to put themselves into what may be viewed as limited or ‘not quite right’ categories. We believe that adding the response option ‘mostly heterosexual’ to the sexual identity question used in the present study likely decreased the risk of response bias; however, some portion of study participants may have been unwilling to accurately report their sexual identity, especially given that the NSHLEW interview was conducted face-to-face. Given the still prevalent heterosexism and biphobia/homophobia in U.S. society it is likely that stigma plays a role in women’s choice of how they define their sexual identity. On the one hand, internalized bi/homophobia or fear of being stigmatized may have led to underestimates of the number of mostly heterosexual women in the general population. On the other hand, some of the women who reported that they were mostly heterosexual may actually have been lesbian or bisexual (but believed that ‘mostly heterosexual’ was a less stigmatized identity), which would result in underestimates of these groups and overestimates of mostly heterosexual women. Until the stigma associated with minority sexual orientation is eliminated this issue is likely not resolvable.

Second, even in this large, nationally representative sample of U.S. women, the number of women who identified as mostly heterosexual was relatively small (unweighted *n* = 72, weighted *n* = 48). Although large enough for the types of statistical analyses used in this paper, the number of mostly heterosexual women is not large enough to delve more deeply into potential subgroup differences (see Implications, below) that may help explain mostly heterosexual women’s elevated risk of substance use/misuse. Third, the sample was interviewed in 2001. Given that research on mostly heterosexuals has developed only recently, there are no historical or longitudinal data that could identify possible change (or lack thereof) in characteristics of mostly heterosexual women in the 14 years since data collection. Finally, the data analyzed here are cross-sectional. Although it seems more likely that a non-heterosexual identity increases risks of substance use/misuse, rather than the reverse, we cannot draw firm conclusions about temporal/causal associations among variables based only on cross-sectional analyses.

Despite these limitations, findings from the study add to the small but growing literature focusing on the health of adult women who do not fit neatly in either the heterosexual or the sexual minority groups that are generally included in such studies. The nationally representative sample and the statistical control for potential confounding variables (age, race/ethnicity, and education) increase confidence in the validity of our findings of elevated risk for alcohol, marijuana, and cocaine use among women who identify themselves as mostly heterosexual.

### Implications

One important implication of our findings, and of studies of younger mostly heterosexual women that also have found elevated rates of health risk behaviors, is that future research should include more nuanced measures of sexual identity – beyond the standard three categories of heterosexual, bisexual, lesbian/gay. Some national surveys (e.g., the National Longitudinal Study of Adolescent Health [Add Health], the Growing Up Today Study [GUTS], and the Australian Longitudinal Study of Women’s Health) offer five sexual identity options, but many surveys still do not. Studies that force women (and men) with intermediate sexual identities to choose among the three traditional options produce results that are biased to an unknown degree by misclassification of sexual identity, thus yielding misleading estimates of health risks of sexual minority subgroups. We suggest that the substantially large group of women who identify as mostly heterosexual has been misclassified in most studies of SMW’s health.

A second implication for future research is the possibility (which we were unable to explore due to sample size limitations) that mostly heterosexual women may not be a homogeneous group. For example, one subgroup may be women who are motivated toward risk-taking and sensation-seeking, which could include experimentation with same-sex sexual relations as well as with alcohol and other substances, and other health-risk behaviors [15]. A second subgroup may be women who are uncertain or conflicted about their sexual identity. Such women could experience intrapersonal discrepancies among the various dimensions of sexual orientation (attraction, behavior, identity) [32] or might experience a discrepancy between intrapersonal feelings of same-sex attraction or identity and the values and norms of a more conservative social environment (e.g., a fundamentalist religion that condemns any form of same-sex feeling or behavior) . A third subgroup might be women who identify as mostly heterosexual as an intermediate identity in transitioning from an only heterosexual identity to a bisexual or lesbian identity (or the reverse) [35–37]. Further research is needed to explore whether such subgroups of mostly heterosexuals can be identified and, if so, to better understand their personal characteristics, social and sexual behavior, and possible health disparities.

Despite a growing body of evidence for increased substance use and other health risk behaviors among mostly heterosexual women, these findings do not appear to have found their way into health care settings. Because mostly heterosexual women generally do not regard themselves as sexual minorities, they are not likely to seek care from sexual minority-specific health care facilities. And they remain invisible in facilities that primarily serve heterosexual populations, where many health care professionals do not ask patients about their sexual identity, and even those who do, typically ask patients only whether they are gay or straight and/or whether they have sex with men, women, or both [38]. Such questions provide women who view themselves as ‘mostly’ heterosexual little opportunity to discuss their sexual orientation, and providers miss potentially important opportunities to educate and counsel.

## Conclusions

As additional knowledge accumulates about the health profiles of mostly heterosexual women, the need becomes critical to educate health and mental health professionals about the existence, large numbers, and characteristics of mostly heterosexual women, so that health care providers can more accurately identify these patients, assess their health risks, and provide appropriate treatment.

 It is striking the extent to which the growing research on mostly heterosexual individuals (including our own) focuses on *negative* health behaviors and adverse health outcomes in this population. One recent, comprehensive, and thoughtful review summarized findings of 60 papers that used data from 22 samples in 5 Western countries [15]. Health outcomes included internalizing disorders (anxiety, depression, suicidality), body image and disordered eating behavior, sexual/reproductive problems, substance use, sexual risk-taking, and other risk-taking. In a short section on “risk and protective factors,” only a handful of studies examined potential protective factors, and we identified only one finding that reflected *positively* on mostly heterosexuals: in one study, boys who identified as mostly heterosexual appeared to have higher scores on religiosity/spirituality, school liking, and school connectedness [39]. Without minimizing the importance of knowledge about increased health risks in mostly heterosexual persons, we suggest that future research should also investigate more positive personality attributes (e.g., tolerance of ambiguity, cognitive and affective flexibility, nonauthoritarianism) as well as other individual resilience factors and environmental protective factors. Indeed, the search for potential *subgroups* suggested earlier might identify one or more subgroups with relatively good physical and mental health and a basic satisfaction with an intermediate sexual identity. Learning more about both the strengths and the vulnerabilities of persons who identify as mostly heterosexual would produce a more complete picture of this interesting and long-neglected population subgroup.

## Abbreviations

SMW: Sexual Minority Women
CSI: Concealable Stigmatized Identities
NSHLEW: National Study of Health and Life Experiences of Women
